# Three Separate Spike Antigen Exposures by COVID-19 Vaccination or SARS-CoV-2 Infection Elicit Strong Humoral Immune Responses in Healthcare Workers

**DOI:** 10.3390/vaccines10071086

**Published:** 2022-07-06

**Authors:** Thomas Theo Brehm, Felix Ullrich, Michelle Thompson, Julia Küchen, Dorothee Schwinge, Anthea Spier, Samuel Huber, Johannes K. Knobloch, Martin Aepfelbacher, Marylyn M. Addo, Ansgar W. Lohse, Marc Lütgehetmann, Julian Schulze zur Wiesch

**Affiliations:** 1 I. Department of Medicine, University Medical Center Hamburg-Eppendorf, Martinistraße 52, 20246 Hamburg, Germany; 6904487@stud.uke.uni-hamburg.de (F.U.); michelle-thompson@t-online.de (M.T.); julia.kuechen@t-online.de (J.K.); d.schwinge@uke.de (D.S.); s.huber@uke.de (S.H.); m.addo@uke.de (M.M.A.); a.lohse@uke.de (A.W.L.); j.schulze-zur-wiesch@uke.de (J.S.z.W.); 2 German Center for Infection Research (DZIF), Partner Site Hamburg-Lübeck-Borstel-Riems, 20246 Hamburg, Germany; j.knobloch@uke.de (J.K.K.); mluetgehetmann@uke.de (M.L.); 3 Institute of Medical Microbiology, Virology and Hygiene, University Medical Center Hamburg-Eppendorf, Martinistraße 52, 20246 Hamburg, Germany; a.spier@uke.de (A.S.); maepfelbacher@uke.de (M.A.); 4 Institute for Infection Research and Vaccine Development, University Medical Center Hamburg-Eppendorf, Martinistraße 52, 20246 Hamburg, Germany

**Keywords:** SARS-CoV-2, COVID-19, healthcare workers, vaccination, mRNA vaccines, immunity, humoral immune response, infection rate

## Abstract

Background: The immunogenicity of different COVID-19 vaccine regimens and combinations in naïve and convalescent individuals has not been formally tested in controlled studies, and real-life observational studies are scarce. Methods: We assessed the SARS-CoV-2 infection and COVID-19 vaccination-induced immunity of 697 hospital workers at the University Medical Center Hamburg-Eppendorf between 17 and 31 January 2022. Results: The overall prevalence of anti-NC-SARS-CoV-2 antibodies indicating prior infection was 9.8% (n = 68) and thus lower than the seroprevalence in the general population. All vaccinated individuals had detectable anti-S1-RBD-SARS-CoV-2 antibodies (median AU/mL [IQR]: 13,891 [8505–23,543]), indicating strong protection against severe COVID-19. Individuals who received three COVID-19 vaccine doses (median AU/mL [IQR]: 13,856 [8635–22,705]) and those who resolved a prior SARS-CoV-2 infection and had received two COVID-19 vaccine doses (median AU/mL [IQR] 13,409 [6934–25,000]) exhibited the strongest humoral immune responses. Conclusions: The current study indicates that three exposures to the viral spike protein by either SARS-CoV-2 infection or COVID-19 vaccination are necessary to elicit particularly strong humoral immune responses, which supports current vaccination recommendations.

## 1. Introduction

Since the onset of the coronavirus disease 2019 (COVID-19) pandemic, we have been conducting a prospective severe acute respiratory syndrome coronavirus type 2 (SARS-CoV-2) seroprevalence study among hospital workers at the University Medical Center Hamburg-Eppendorf in Hamburg, Germany. Previously, we reported on the seroprevalence during the first months of the pandemic at several time points between March and July 2020 [1] and at the end of the third wave in May 2021 [2]. We longitudinally assessed the prevalence of antibodies directed against the receptor-binding domain (RBD) of the viral spike protein (S), which are elicited both by SARS-CoV-2-infection and COVID-19 vaccination and of antibodies directed against the viral nucleocapsid (NC), which are only present after natural infection. Anti-NC-SARS-CoV-2 may, therefore, serve as a surrogate marker for the SARS-CoV-2 infection rate among our cohort. In May 2021, we could demonstrate a relatively low SARS-CoV-2 infection rate of 4.7%. In the meantime, there was an unprecedented surge in SARS-CoV-2 infections in Germany in fall 2021, driven by seasonality, the emergence of the delta variant (B.1.617.2), and, more recently, the omicron variant (B.1.1.529) [3]. Those variants were shown to have a higher transmissibility rate compared with the ancestral strain, which has raised concerns about nosocomial SARS-CoV-2 transmission [4,5]. We, therefore, aimed to assess the prevalence of anti-NC-SARS-CoV-2 antibodies among our study cohort to determine the effectiveness of infection control interventions in preventing nosocomial transmission of delta and omicron SARS-CoV-2 variants.

At the last study visit, we demonstrated solid COVID-19 vaccine-induced humoral immune responses when most study participants had received primary vaccination with two vaccine doses. In the meantime, it has been shown that vaccine-induced immunity wanes over time and that these immune responses have reduced effectiveness against the novel SARS-CoV-2 variants. In response, the administration of the third dose of an mRNA COVID-19 vaccine dose, also known as a booster dose, was recommended in Germany in prioritized groups, including healthcare workers, in October 2021 [6] and for the general population in December 2021 [7]. As of January 2022, the population uptake of two doses of COVID-19 vaccines in Germany was 74.0%. At the same time, 53.1% had received an additional booster dose [8]. After adenovirus-vector vaccine-related thromboembolic issues were reported, the administration of AZD1222 (Vaxzevria^®^, AstraZeneca) was only continued in vaccinees older than 60 years in Germany in March 2021. With this policy change, many healthcare workers in Germany received a heterologous primary vaccination with one dose of AZD1222 followed by either BNT162b2 (Comirnaty^®^, BioNTech/Pfizer) or mRNA-1273 (Spikevax^®^, Moderna) as the second dose. Previously, studies by us and others have suggested that this heterologous primary vaccination with AZD1222 followed by an mRNA vaccine may confer more potent humoral immune responses than a homologous vaccination regimen [2,9,10,11]. Due to these findings and the advantage of a shorter time interval between vaccine doses, German national guidelines now recommend the administration of an mRNA vaccine for all vaccinees who had received one dose of AZD1222 regardless of age [12]. Since only mRNA vaccines are currently recommended as third vaccine doses, many individuals with homologous primary vaccination with AZD1222 have now received heterologous boosting with either BNT162b2 or mRNA-1273. The immunogenicity of the various possible COVID-19 vaccine combinations in naïve and convalescent individuals who had knowingly or unknowingly contracted SARS-CoV-2 has not been formally tested in controlled studies and real-life observational studies comparing immunogenicity are scarce. To assess the SARS-CoV-2 infection and COVID-19 vaccination-induced immunity among employees of the University Medical Center Hamburg-Eppendorf, we performed another study visit between 17 and 31 January 2022 as part of our ongoing seroprevalence study.

## 2. Materials and Methods

Hospital workers of the University Medical Center Hamburg-Eppendorf in Hamburg, Germany, participating in our ongoing longitudinal study, were invited to provide a serum sample between 17 and 31 January 2022. Prior SARS-CoV-2 infections and COVID-19 vaccination status were assessed using an online REDcap electronic data capture tool that had been specifically designed for the present study.

The study protocol was reviewed and approved by the Ethics Committee of the Medical Council of Hamburg (PV 7298), and written informed consent was obtained by all study participants before recruitment. Due to the small sample size, the two currently licensed mRNA vaccines, BNT162b2 and mRNA-1273, are subsequently subsumed into the single category “mRNA vaccines.”

Antibodies against the receptor-binding domain (RBD) domain of the viral spike protein (S) were detected with the quantitative Elecsys anti-S1-RBD-SARS-CoV-2 assay (Roche, Mannheim, Germany; cut off 0.8 AU/mL), which has a reported sensitivity of 99.8% and a specificity of 100% [13]. In addition, antibodies against the viral nucleocapsid protein (NC) were detected with the qualitative Elecsys anti-NC-SARS-CoV-2 Ig assay (Roche, Mannheim Germany; cut off ≥1 COI/mL), which has a reported sensitivity and specificity of 99.5% and 99.8%, respectively [14]. We used anti-S1-RBD-SARS-CoV-2 antibodies to quantify the humoral immune response to COVID-19 vaccination and used anti-NC-SARS-CoV-2 antibodies s as a surrogate marker for SARS-CoV-2 infection rate since they are only present after natural infection.

Both electrochemiluminescence immunoassays (Cobas e411, Roche; Mannheim, Germany) use a double-antigen sandwich assay format that detects both IgA, IgM, and IgG. Samples with titers above 250 U/mL were manually diluted 1:100 in dilution buffer according to the manufacturer’s recommendations to increase the linear range to 25,000 U/mL.

A two-tailed Mann-Whitney U test was used to analyze median antibody titers between two subgroups, and Kruskal-Wallis was used to analyze median antibody titers between more than two subgroups. A general linear model was calculated among participants with three vaccine doses to predict the anti-S1-RBD-SARS-CoV-2 antibody titer from sex, age, different vaccination regimen, time since the booster vaccination, and presence of anti-NC-SARS-CoV-2 antibodies. Statistical analyses were performed using GraphPad Prism, version 9 for macOS (GraphPad Software, La Jolla, CA, USA) and SPSS, version 26 for macOS (IBM Corporation, Armonk, NY, USA). *p* values less than 0.05 were considered statistically significant.

## 3. Results

### 3.1. Characterization of the Study Population

Between January 17 and 31, 2022, 697 hospital workers participated in the current study visit, representing around 6% of all employees of the University Medical Center Hamburg-Eppendorf. The median age of the current study cohort age was 40 years (interquartile range [IQR] 31–50 years) and 78.5% (n = 547) of the participants were women (Table 1).

Most study participants were healthcare workers directly involved in patient care at different departments of our tertiary care center: 37.4% were nurses (n = 261), 20.1% were physicians (n = 140), 16.1% were medical technicians (n = 112), and 12.2% (n = 85) had other professions The remaining 14.2% (n = 99) were other employees not directly involved in patient care. Even though we did not recruit a strictly representative sample of employees at our hospital, the relative proportion of different professional groups in our study cohort closely resembled the overall distribution of different professional groups at our hospital (30.1% nurses, 25.8% medical doctors, 44.1% others). Most study participants had received three vaccine doses (n = 644; 92.4%), fewer were vaccinated once (n = 5; 0.7%), twice (n = 38; 5.5%) or four times (n = 5; 0.7%). Five study participants (0.7%) had not yet been vaccinated at this current study time point. The various different vaccination regimens are shown in Table 1.

### 3.2. Serological Results

**Figure 1:**
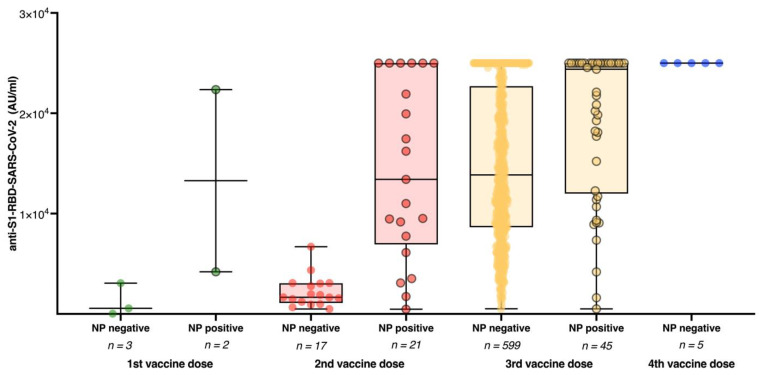
Anti-S1-RBD-SARS-CoV-2 antibody titers stratified by infection status and different vaccination regimens. Anti-S1-RBD-SARS-CoV-2 antibody titers among study participants with (NP positive; dots with black edging) and without (NP negative; dots without black edging) detectable anti-NC-SARS-CoV-2 based on their vaccination status.

All 692 (99.3%) study participants who had received at least one dose of a COVID-19 vaccine had detectable anti-S1-RBD-SARS-CoV-2 antibodies; the median titer was 13,891 AU/mL (IQR 8505–23,528 AU/mL). The rate of individuals with anti-S1-RBD-SARS-CoV-2 antibody titers higher than 10,000 AU/mL increased from 8.3% (n = 72) at the last study visit in May 2021 to 65.9% (n = 459) at the current study visit. Anti-NC-SARS-CoV-2 antibodies indicating prior SARS-CoV-2 infection were now detected in 9.8% (n = 68) individuals. Study participants with and without detectable anti-NC-SARS-CoV-2 antibodies did not significantly differ in median age and sex, and the rate of individuals with detectable anti-NC-SARS-CoV-2 antibodies was similar among different professional groups (Supplemental Table S1). Among study participants without prior SARS-CoV-2 infection reflected by the absence of anti-NC-SARS-CoV-2 antibodies, the median anti-S1-RBD-SARS-CoV-2 antibody titer significantly increased between those who received only one (median AU/mL [IQR]: 564 [24–3070]) to those who received two (median AU/mL [IQR]: 1663 [1094–3060]), three (median AU/mL [IQR]: 13,856 [8,635–22,705]) or four (median AU/mL [IQR]: 25,000 [25,000–25,000]) COVID-19 vaccine doses (*p* < 0.0001) (Figure 1).

**Figure 2:**
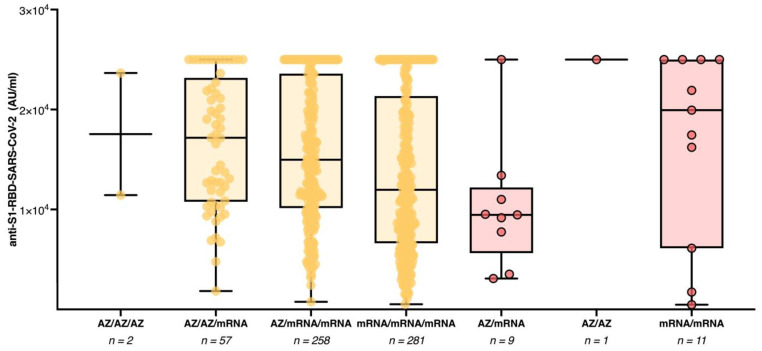
Anti-S1-RBD-SARS-CoV-2 antibody titers among study participants with three separate spike antigen exposures by COVID-19 booster vaccination or SARS-CoV-2 infection. Anti-S1-RBD-SARS-CoV-2 antibody titers among study participants with three vaccine doses and no detectable anti-NC-SARS-CoV-2 antibodies (yellow) or two vaccine doses with detectable anti-NC-SARS-CoV-2 antibodies (red).

Study participants who previously resolved a SARS-CoV-2 infection had significantly higher median anti-S1-RBD-SARS-CoV-2 antibody titers in the respective subgroups with two (median AU/mL [IQR] 13,409 [6934–25,000] vs. 1663 [1094–3060]; *p* < 0.0001) and three (median AU/mL [IQR]: 24,393 [11,991–25,000] vs. 13,856 [8635–22,705]; *p* < 0.0001) COVID-19 vaccine doses. None of the five individuals who had received four vaccine doses had detectable anti-NC-SARS-CoV-2 antibodies. Therefore no statistical analysis was performed for this subgroup. We further compared anti-S1-RBD-SARS-CoV-2 antibody titers among anti-NC-SARS-CoV-2 antibody-negative study participants with three homologous versus heterologous COVID-19 vaccine doses (Figure 2).

Those study participants who had received a heterologous vaccination regimen including both AZD1222 and mRNA vaccines (median AU/mL [IQR]: 15,105 [10,296–23,532]) had higher median antibody titers than those who received three mRNA vaccine doses (median AU/mL [IQR]: 11,973 [6618–21,354]; *p* < 0.0001). Among the study participants who had received a heterologous COVID-19 vaccination regimen, no difference in immune response was observed between those with the vaccination regimen AZD1222/mRNA/mRNA (median AU/mL [IQR]: 14,980 [10,140–23,595]) compared to those with the vaccination regimen AZD1222/AZD1222/mRNA (median AU/mL [IQR]: 17,171 [10,776–23,165]; *p* = 3.6). Median antibody titers for all different subgroups are provided in Supplemental Table S2. The results of the general linear model revealed that among study participants with three vaccine doses (n = 644), age, sex, vaccination regimen, time since the booster vaccination, and presence of anti-NC-SARS-CoV-2 antibodies significantly predicted the anti-S1-RBD-SARS-CoV-2 antibody titer (F = 11.1; *p* < 0.001). Only the time since the booster vaccination (F = 125.2; *p* < 0.001) and the presence of anti-NC-SARS-CoV-2 antibodies (F = 6.7; *p* = 0.01) added statistically significantly to the prediction. Age, sex, and vaccination regimen were not found to be significant predictors of anti-S1-RBD-SARS-CoV-2 antibody titers (*p* > 0.05). We further analyzed the kinetics of anti-S1-RBD-SARS-CoV-2 antibody titers among study participants without prior SARS-CoV-2 infection between the last study visit in May 2021 and the current study visit (Supplemental Figure S1). All but one of the 206 study participants who had received one COVID-19 vaccine dose and who participated in the last study visit in May 2021 and two additional COVID-19 vaccine doses up to the present study visit demonstrated an increase in the anti-S1-RBD-SARS-CoV-2 antibody titer (median AU/mL [IQR]: 60 [29–120] vs. 17,614 [11,538–25,000]; *p* < 0.0001). Likewise, the vast majority (n = 222, 88.4%) of the 251 study participants who were measured after two COVID-19 vaccine doses at the last study visit and now had three COVID-19 vaccine doses at the present study visit showed a further increase in the humoral immune responses. In comparison, 26 (10.4%) healthcare workers had a decrease in antibody titers and three had persistently high antibody titers at the highest measurable concentration (median AU/mL [IQR]: 1622 [692–5039] vs. 11,780 [6725–19,497]; *p* < 0.0001).

## 4. Discussion

We describe the SARS-CoV-2 infection and COVID-19 vaccination-induced immunity of 697 hospital workers at the University Medical Center Hamburg-Eppendorf between 17 and 31 January 2022.

One of our key findings is that the prevalence of anti-NC-SARS-CoV-2 antibodies, which can serve as a surrogate parameter for prior infection, was 9.8% at the current study visit. While several seroprevalence studies have reported on the infection rates in healthcare workers throughout the early phases of the COVID-19 pandemic, data generated at later phases of the pandemic during which the delta and omicron variants, which have been reported to be more transmissible than previous strains, have been circulating are very scarce. The prevalence of anti-NC-SARS-CoV-2 represents a doubling of the SARS-CoV-2 infection rate in our study cohort since the last study visit in May 2021. Of note, the official number of recovered COVID-19 patients in the city-state of Hamburg was 262,907 by the end of January 2022, which represents 14.2% of all inhabitants, assuming that no one had been infected more than once. While, on the one hand, some inhabitants certainly were infected more than once, on the other hand, further unidentified or unreported COVID-19 cases must be assumed. In summary, the SARS-CoV-2 incidence rate among hospital workers in our study appears somewhat lower than in the general population, confirming that established infection control interventions remained effective in preventing nosocomial SARS-CoV-2 transmissions when the delta and omicron variants are the predominant circulating variants in the first quarter of 2022.

From our data, we conclude that a total of three spike antigen exposures by booster COVID-19 vaccination or any combination of SARS-CoV-2 infection with two vaccinations are necessary to achieve high antibody levels reliably. This supports current vaccination recommendations and is in line with previous studies demonstrating that triple-vaccinated naive individuals reach comparable levels of neutralization capacity against the omicron variant as vaccinated convalescents and twice-vaccinated individuals contracting break-through infections [15]. Likewise, efficacy studies showed that protection against SARS-CoV-2 infection in twice-vaccinated individuals considerably wanes after six months, whereas convalescents boosted with two vaccine doses and triple-vaccinated individuals are protected for 12 months [16,17]. Also, previous studies have shown that a single SARS-CoV-2 infection does not provide the same neutralization capacity as a combination of infection and vaccination [15]. Our findings are in line with previous investigations, which have shown that all licensed vaccines given as a homologous or heterologous third booster dose are effective in enhancing neutralizing antibody and cellular immune responses, notwithstanding which vaccines had been received in the initial vaccine course [18]. In our study, the administration of a booster vaccine resulted in high antibody levels in the majority of study participants regardless of the primary vaccination regimen, with a more than 7-fold increase between the second and the third vaccine dose. While among individuals with three vaccine doses, those with heterologous vaccination regimen had higher anti-S1-RBD-SARS-CoV-2 antibody levels among those compared to those who received three doses of an mRNA vaccine, multiple linear regression analysis revealed that only time since the booster vaccination and presence of anti-NC-SARS-CoV-2 antibodies, but not age, sex, and vaccination regimen significant predictors of anti-S1-RBD-SARS-CoV-2 antibody titers. Our study is subject to limitations. As described above, we did not recruit a strictly representative sample of hospital employees at our institution, which may limit the generalizability of the results. Since employees who have been vaccinated may be more likely to participate in our study, we cannot reliably assess the COVID-19 vaccination uptake among hospital workers at our institution. Also, employees with current SARS-CoV-2 infections may not have been able to participate in the current study visit, so we may underestimate the infection rate in our cohort to some degree. While 1253 and 872 hospital workers respectively participated in the last follow-up visits in June 2020 and May 2021, only 697 individuals participated in the current study visit. As in any real-world observational study, the study participants were not randomly assigned to different COVID-19 vaccination regimens, and confounding may be expected due to dissimilarities in the different subgroups and differences in the time intervals between the respective vaccine doses and between the last vaccination and the current study visit. Moreover, given the limited sample size since we subsumed the mRNA vaccines BNT162b2 and mRNA-1273 to one single category and are therefore not able to assess potential differences in immunogenicity amongst different vaccination regimens with one or both of those vaccines. An additional limitation is that we quantitatively assessed the concentration of anti-SARS-CoV-2 antibodies but not their neutralizing capacity or cellular immune responses. While neutralizing antibody levels are highly predictive of immune protection from symptomatic SARS-CoV-2 infection [19], the sheer concentration of antigen-specific antibodies may not be a reliable surrogate and vaccine-induced cellular immune responses have been demonstrated to remarkably contribute to protection from severe disease [20]. Lastly, we did not assess any data on potential side effects of COVID-19 vaccines in our study cohort.

Since the beginning of the pandemic, a combination of non-pharmaceutical interventions like social distancing, masking, testing, and contact tracing and the rollout of COVID-19 vaccines have been successful in mitigating the spread of SARS-CoV-2. While COVID-19 vaccines are highly efficient in preventing severe disease and reducing SARS-CoV-2-related morbidity and mortality, the SARS-CoV-2 case numbers have spiked all over the world despite large-scale immunization campaigns. Therefore, adherence to non-pharmaceutical interventions will remain crucial throughout the evolution of the COVID-19 pandemic to protect both healthcare workers and vulnerable patient groups. This is even more important in light of the expected renewed increase in influenza numbers and the cocirculation of both SARS-CoV-2 and seasonal influenza viruses in the northern hemisphere in the upcoming influenza season. While we show that three exposures to the viral spike protein by either SARS-CoV-2 infection or COVID-19 vaccination elicit robust humoral immune responses, prospective longitudinal studies will be needed to investigate the duration of immune protection from circulating SARS-CoV-2 variants after COVID-19 vaccination, assess the necessity for novel omicron-targeted vaccines, and determine the timing for additional booster vaccinations.

## 5. Conclusions

Our study indicates that three exposures to the SARS-CoV-2 spike protein by either infection or vaccination are necessary to elicit particularly strong humoral immune responses, which supports the current COVID-19 vaccination recommendations.

## Figures and Tables

**Table 1:** Characterization of the study population.

Sex	n (%)
Male	143 (20.5)
Female	547 (78.5)
Diverse	7 (1.0)
**Age**	
Median	40
IQR	31–50
**Profession**	
Nurse	261 (37.4)
Physician	140 (20.1)
Medical Technician	112 (16.1)
Other healthcare worker	85 (12.2)
Non-healthcare worker	99 (14.2)
**COVID-19 Vaccine Doses**	
**0**	5 (0.7)
**1**	5 (0.7)
*Ad26.COV2.S, n*	3 (0.4)
*mRNA, n*	2 (0.3)
**2**	38 (5.5)
*AZD1222/AZD1222, n*	1 (0.2)
*AZD1222/mRNA, n*	12 (1.7)
*mRNA/mRNA, n*	25 (3.6)
**3**	644 (92.4)
*AZD1222/AZD1222/AZD1222, n*	2 (0.3)
*AZD1222/AZD1222/mRNA, n*	60 (8.6)
*AZD1222/mRNA/mRNA, n*	276 (39.6)
*mRNA/mRNA/mRNA, n*	303 (43.5)
*N/A, n*	3 (0.4)
**4**	5 (0.7)
*AZD1222/mRNA/mRNA/mRNA, n*	1 (0.2)
*mRNA/mRNA/mRNA/mRNA, n*	4 (0.6)

## Data Availability

Not applicable.

## Supplementary Materials

The following supporting information can be downloaded at: https://www.mdpi.com/article/10.3390/vaccines10071086/s1, Figure S1: Anti-S1-RBD-SARS-CoV-2 antibody titers over time; Table S1: Characteristics of anti-NC negative and anti-NC positive study participants, Table S2. Median antibody titers stratified by different vaccination regimens
