# Supplementary material for: Three Separate Spike Antigen Exposures by COVID-19 Vaccination or SARS-CoV-2 Infection Elicit Strong Humoral Immune Responses in Healthcare Workers
Source: Vaccines (Basel). 2022 Jul 6;10(7):1086. doi: 10.3390/vaccines10071086 (PMC9317472; doi:10.3390/vaccines10071086)
Supplement: Supplementary file 1 [file vaccines-10-01086-s001.zip › vaccines-1746553-supplementary.pdf]

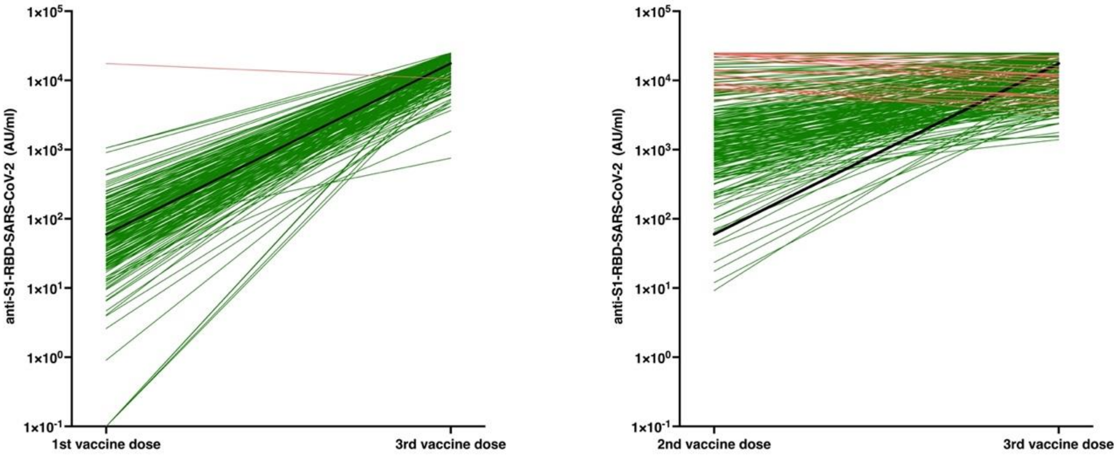

Figure S1. Anti-S1-RBD-SARS-CoV-2 antibody titers over time

Anti-S1-RBD-SARS-CoV-2 antibody titers are shown for study participants without anti-NC-SARS-CoV-2 antibodies indicating prior SARS-CoV-2 infection between the last study visit in May 2021 and the current study visit. The left panel shows the antibody titers for those who had received one vaccine dose in May 2021 and three vaccine doses by now. The right panel shows antibody titers for those who had received two vaccine dose in May 2021 and three vaccine doses by now. Increasing titers are portrayed as green lines, decreasing titers are portrayed as red lines. The fat black line connects the median titers of the two study visits.

Table S1. Characteristics of anti-NC negative and anti-NC positive study participants

	**anti-NC negative (n=629)**	**anti-NC positive (n=68)**
**Sex**		
Male, n(%)	131 (20.8)	12 (17.6)
Female, n(%)	491 (78.1)	56 (82.4)
Diverse, n(%)	7 (1.1)	0
**Age**		
Median	40	35
IQR	31; 51	28; 48
**Profession**		
Nurse, n(%)	234 (37.2)	27 (39.7)
Physician, n(%)	130 (20.7)	10 (14.7)
Medical Technician, n(%)	100 (15.9)	12 (17.6)
Other heathcare worker, n(%)	75 (11.9)	10 (14.7)
Non-healthcare worker, n(%)	90 (14.3)	9 (13.2)

Table S2. Median antibody titers stratified by different vaccination regimens

COVID-19 vaccine doses	n	Age	Female	anti-NC positive	anti-S1-RBD (total)	anti-S1-RBD (anti-NC positive)	anti-S1-RBD (anti-NC negative)
		Median (IQR)	*N (%)*	*N (%)*	*Median (IQR)*	*Median (IQR)*	*Median (IQR)*
**0**	5	46 (44; 49)	4 (80)	0	<0.4	N/A	<0.4
**1**	5	59 (56; 60)	4 (80)	2 (40)	3070 (429; 3’354)	22’357 (8’740; 17’818)	564 (24; 3070)
Ad26.COV2.S	3	59 (58; 63)	2 (67)	0	564 (294; 1’817)	N/A	564 (24; 3070)
mRNA	2	57 (56; 59)	2 (100)	2 (100)	13279 (8’749; 17’818)	13’279 (8’749; 17’818)	N/A
**2**	38	37 (30; 44)	33 (87)	21 (55)	3’944 (1’683; 13’409)	13’409 (6934; 25’000)	1663 (1’094; 3’060)
AZD1222/AZD1222	1	54	1 (100)	1 (100)	25’000	25’000	N/A
AZD1222/mRNA	12	35 (27; 45)	12 (100)	9 (75)	8458 (3’078; 9’487)	9’461 (7’747; 9’887)	658 (677; 617)
mRNA/mRNA	25	36 (31; 44)	20 (80)	11 (44)	3’082 (1’652; 16’528)	19’941 (11’170; 25’000)	1’777 (1’614; 2’776)
**3**	644	40 (31; 51)	504 (78)	45 (7)	14’442 (8’847; 23’587)	24’393 (11’991; 25’000)	13’856 (8’635; 22’705)
AZD1222/AZD1222/AZD1222	2	51 (48; 55)	2 (100)	0	17’545 (14’491 – 20’600)	N/A	17’545 (14’491 – 20’600)
AZD1222/AZD1222/mRNA	60	55 (45; 58)	49 (82)	3 (5)	19’062 (12’657; 25’000)	20’215 (20’025; 22’608)	17’171 (10’776; 23’165)
AZD1222/mRNA/mRNA	276	40 (31; 52)	217 (79)	18 (7)	15’269 (10’270; 24’330)	22’130 (12’588; 25’000)	14’980 (10’140; 23’595)
mRNA/mRNA/mRNA	303	37 (29; 47)	233 (77)	22 (7)	12’269 (6’857; 22’875)	24’480 (13’758; 25’000)	11’973 (6’618; 21’354)
**4**	5	30 (27; 36)	2 (40)	0	25’000	N/A	25’000
AZD1222/mRNA/mRNA/mRNA	1	30	0	0	25’000	N/A	25’000
mRNA/mRNA/mRNA/mRNA	4	31 (27; 38)	2 (50)	0	25’000	N/A	25’000
